# What do spring migrants reveal about sex and host selection in the melon aphid?

**DOI:** 10.1186/1471-2148-12-47

**Published:** 2012-04-03

**Authors:** Sophie Thomas, Nathalie Boissot, Flavie Vanlerberghe-Masutti

**Affiliations:** 1 INRA, UR1052, Génétique et Amélioration des Fruits et Légumes, B.P. 94, F-84143 Montfavet cedex, France; 2 INRA, UMR1062 CBGP, F-34988 Montferrier-sur-Lez, France

## Abstract

**Background:**

Host plants exert considerable selective pressure on aphids because the plants constitute their feeding, mating and oviposition sites. Therefore, host specialisation in aphids evolves through selection of the behavioural and chemical mechanisms of host-plant location and recognition, and through metabolic adaptation to the phloem content of the host plant. How these adaptive traits evolve in an aphid species depends on the complexity of the annual life cycle of that species. The purpose of this field study was to determine how winged spring-migrant populations contribute to the evolution and maintenance of host specialisation in *Aphis gossypii *through host-plant choice and acceptance. We also assessed whether host-specialised genotypes corresponded exclusively to anholocyclic lineages regardless of the environmental conditions.

**Results:**

The spring populations of cotton-melon aphids visiting newly planted melon crops exhibited an unexpectedly high level of genetic diversity that contrasted with the very low diversity characterising the host-specialised populations of this aphid species. This study illustrated *in natura *host-plant-selection pressure by showing the great differences in genetic diversity between the spring-migrant populations (alate aphids) and the melon-infesting populations (the apterous offspring of the alate aphids). Moreover, an analysis of the genetic composition of these alate and apterous populations in four geographic regions suggested differences in life-history strategies, such as host choice and reproductive mode, and questioned the common assertion that *A. gossypii *is an anholocyclic species throughout its distribution area, including Europe.

**Conclusions:**

Our results clearly demonstrate that the melon plant acts as a selective filter against the reproduction of non-specialised individuals. We showed that olfactory cues are unlikely to be decisive *in natura *for host recognition by spring-migrant aphid populations that are not specialised on Cucurbitaceae. The agroecosystem structure and history of the four studied regions may have partially shaped the genetic structure of the spring-migrant populations of *A. gossypii. Cucurbitaceae*-specialised genotypes corresponded exclusively to anholocyclic lineages, regardless of the environmental conditions. However, some genotypes that were genetically close to the host-specialised genotypes and some genotypes that probably originated from wild plants had never been previously sampled; both were holocylic.

## Background

The predictability and abundance of host resources is often assumed to favour ecological specialisation in populations of parasites that have a large spectrum of possible hosts through the disruptive selection of host preference, leading gradually to reproductive isolation and speciation if assortative mating occurs [1-6]. Host-associated biotypes are well documented in plant-feeding insects, and many examples of host races have been described in phytophagous insects in the last ten years. As reviewed by Dres and Mallet (2002) [4], host races are defined as sets of populations of the same species that use different host taxa in the wild, exhibit host fidelity, coexist in sympatry and correspond to separated genetic clusters among which gene flow still occurs. Moreover, these populations usually display a correlation between host choice and mate choice, have a higher fitness on natal than on alternative hosts and produce hybrids that are less fit than the parental forms. Host race status has been especially discussed within the Aphididae [7]. In the case of aphids, host plants exert considerable selective pressure because the plants constitute the aphids' feeding, mating and oviposition sites. Therefore, host specialisation in aphids evolves through the selection of behavioural and chemical mechanisms of host-plant location and recognition as well as through metabolic adaptation to the phloem content of the host plant. How these adaptive traits evolve in an aphid species depends on the complexity of the aphids' annual life cycle.

Like many parasites, aphids have evolved complex life cycles, with successive generations of individuals often specialised to cope with different ecological conditions. However, aphids are among the few organisms that show plasticity in their reproductive mode as an adaptive response to seasonal changes [8]. The typical aphid annual life cycle, called holocycly or cyclical parthenogenesis, includes a single sexual generation in the autumn composed of males and mating females (oviparae) that lay resting eggs. In the spring, a wingless (apterous) female (fundatrix) hatches from the egg and produces other parthenogenetic females (viviparae). The number of successive viviparae generations in the spring and summer depends on environmental factors. All of the descendants of a single fundatrix are genotypically identical and constitute a clone, although several phenotypes are expressed. Indeed, the viviparae may be alate or apterous in response to environmental conditions, with development of alate forms stimulated in crowded colonies. Finally, in the autumn, in response to predictive environmental conditions, the viviparae will produce sexual morphs. Approximately 90% of aphid species are monoecious, *i.e*., they remain on the same host species or closely related plant species throughout their life cycle [9]. In the remaining 10%, which are heteroecious, the aphids alternate between the primary host, usually a woody plant, onto which they migrate during autumn for sexual reproduction, and secondary hosts, usually herbaceous plants, onto which they migrate during spring and develop numerous parthenogenetic colonies. Under specific circumstances, some aphids do not complete such a life cycle and reproduce parthenogenetically throughout the year on herbaceous plants. This life cycle is termed anholocycly. Approximately 3% of all aphid species are entirely anholocyclic throughout their range, and it has been suggested that 30% to 50% of holocyclic aphid species display a variation in their reproductive mode between and even within populations [10].

Only 5% of aphid species are polyphagous [11], comprising crop-pest species that frequently encompass host races or host-specialised populations with a narrow host range. Such patterns have been described for the pea aphid *Acyrthosiphon pisum *[12-14] and the grain aphid *Sitobion avenae *[15,16], which are monoecious with a host-plant range restricted to Fabaceae and Poaceae, respectively. Therefore, ecological specialisation can evolve through feeding behaviour that determines both the host choice - and thus the pool of potential mates - and performance on different host plants [17]. Conversely, the evolution of host races in heteroecious (host-alternating) aphid species may represent a paradox because lineages that differ in their preferences for secondary hosts become admixed at mating sites, *i.e*., on the primary host. In this case, specialisation could evolve through a reinforcement process whereby selection against hybrids - due to host-associated genetic trade-offs - can drive the increase of assortative mating [18]. Host-specialised populations were detected in two heteroecious pest aphid species, the peach-potato aphid *Myzus persicae *[19-21] and the cotton-melon aphid *Aphis gossypii *[22]. These species have a large spectrum of secondary hosts that belong to numerous plant families, among which cultivated crop species represent a very abundant resource [23]. In *M. persicae*, host specialisation was evidenced for anholocyclic and holocyclic lineages, and assortative mating seemed to be promoted by differences in the pattern of sexual activity of both the males and mating females [24]. In *A. gossypii*, the specialised populations consisted of a few anholocyclic lineages [22,25,26], suggesting that host-plant specialisation evolved through the loss of the sexual generation in the life cycle [27].

The cotton-melon aphid *A. gossypii *is distributed worldwide and is found in both tropical and subtropical areas and in temperate zones. This species has been detected on more than 900 plant species [28] and in biotopes affected by human activity [29]. Throughout most of its distribution area, including Europe, the species is considered to be anholocyclic [30,31]. However, in areas with rigorous winters in Japan, China, Korea, India and the United States, a sexual generation has been described on a limited number of primary hosts, including *Hibiscus syriacus, Catalpa bignoïdes *and other ligneous plants (reviewed in [32]). Sexual reproduction on *H. syriacus *has also been reported in Italy [33]. Genetic studies of *A. gossypii *populations collected from large apterous colonies sampled in heavily infested crops all over the world revealed a structure in host races on *Cucurbitaceae*, cotton, *Solanum *and pepper plants. Each host race was dominated by a few asexual clones, probably because of host selection, clone competition and pest-management practices [22,34,35]. Plant-transfer experiments confirmed the existence of host-associated trade-offs in these specialised clones of *A. gossypii*. Moreover, both the genetic and experimental data suggested that plants of the genus *Hibiscus *may be used as refuge by the specialised clones when the seasonal plant resource is no longer available [22].

The aim of this field study was to investigate how winged spring-migrant populations may contribute to the evolution and maintenance of host specialisation in *A. gossypii *through host-plant selection. We also assessed whether host-specialised genotypes corresponded exclusively to anholocyclic lineages in relation to the environmental conditions. The sampling was completed in areas where the studied agrosystems were dominated or not dominated by *Cucurbitaceae *(melon) crops. The host-plant selection and reproductive mode were evidenced from a comparison of the genetic structure of the alate spring-migrant populations that alighted on melon crops with the structure of the apterous populations that consisted of the offspring of the migrants.

## Results

### Genetic diversity of the *Aphis gossypii *populations in melon crops

**Figure 1:**
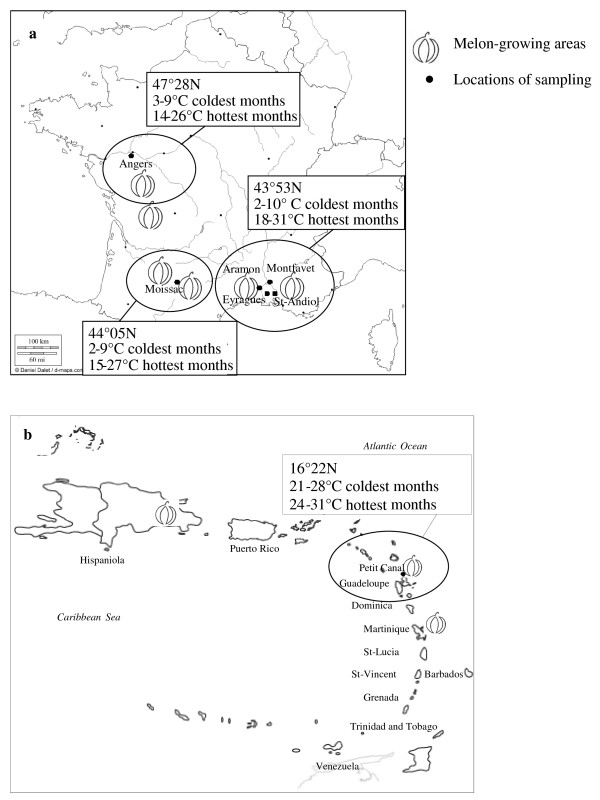
**Melon-growing areas and aphid-sampling sites in a. France and b. the Lesser Antilles**.

To investigate the genetic diversity of *A. gossypii*, we sampled alate and apterous aphids from 2004 to 2009 in four locations in the southeast (SE) of France, one location in the southwest (SW) and west (W) of France and one location in the Lesser Antilles (LA) (Figure 1). The aphids were genotyped using eight microsatellite markers.

Among the 4792 aphids analysed, we discriminated 596 multilocus genotypes (MLGs). The majority of the MLGs were present as a single copy, and only four of the MLGs had a frequency higher than 5% of the total number of individual genotypes (Table 1). We discriminated 413 MLGs among the 1868 alate aphids; 18 to 189 of the alate MLGs were characterised in the populations from the different growing areas. In the alate aphid samples, the allelic diversity ranged from 9 to 49 alleles per locus, with 203 alleles identified across all of the loci. We discriminated 280 MLGs among the 2924 apterous aphids; three to 136 of the apterous MLGs were characterised in the populations from the different growing areas. In the apterous aphid samples, the allelic diversity ranged from 9 to 44 alleles per locus, with 165 alleles identified across all of the loci.

**Table 1:** Information about studied *A. gossypii *populations

	Total	Total Al	Total Ap	Southeast of France		Southwest of France		West of France		Lesser Antilles	
				Al	Ap	Al	Ap	Al	Ap	Al	Ap
N individuals	4792	1868	2924	1148	1540	200	402	173	260	347	722
N alleles	226	203	165	119	108	117	72	140	134	69	20
G	596	413	280	189	136	84	41	136	128	18	3
g_1 _(> 5%)	4	5	6	4	4	4	4	1	3	1	1
g_2 _(< 5%)	162	93	84	61	59	8	16	20	18	6	1
g_3 _(single)	430	315	190	124	73	72	21	115	107	11	1

Number of individuals (N), number of multilocus genotypes (G), number of multilocus genotypes when the frequencies were higher than 5% (g_1_) and when the frequencies were lower than 5% (g_2_), and the number of unique MLGs (g_3_), in the alate (Al) and apterous (Ap) *A. gossypii *sampled in France and the Lesser Antilles.

**Figure 2:**
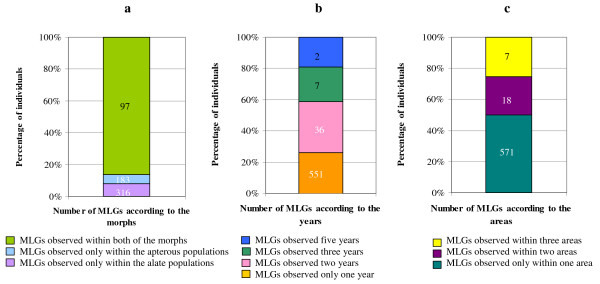
**Occurrence of multilocus genotypes in the *A. gossypii *populations sampled in France and Lesser Antilles**. The numbers of multilocus genotypes are shown in the bars and the percentages of individuals exhibiting those multilocus genotypes are shown in the axis y according to a. morphs, b. years and c. areas.

Among the 596 MLGs discriminated, 316 were observed within only the alate samples, 183 were observed within only the apterous samples and 97 were observed within both of the morphs (Figure 2a). The distribution was different for the numbers of individuals: 8% of the individuals had an MLG that was observed within only the alate samples, 6% of the individuals had an MLG that was observed within only the apterous samples and 86% of individuals had an MLG that was observed within both the alate and apterous samples. We observed 551 MLGs that were present in only one of the studied years, but more than 70% of the individuals had an MLG that was observed in two or more years (Figure 2b). We observed 571 MLGs that were present in only one of the areas, but approximately 50% of the individuals had an MLG that was observed in two or more areas (Figure 2c). None of the MLGs that were observed in the LA were observed in the other areas.

### Genotypic diversity of the alate populations vs. the apterous populations

Among each morph, the genotypic diversity of the samples was investigated at three levels: within an area, between years and between areas. Three diversity indexes were considered, reflecting the clonal richness, *R *(number of clones relative to the sample size); the clonal diversity, D*; and the clonal evenness, *V*.

**Figure 3:**
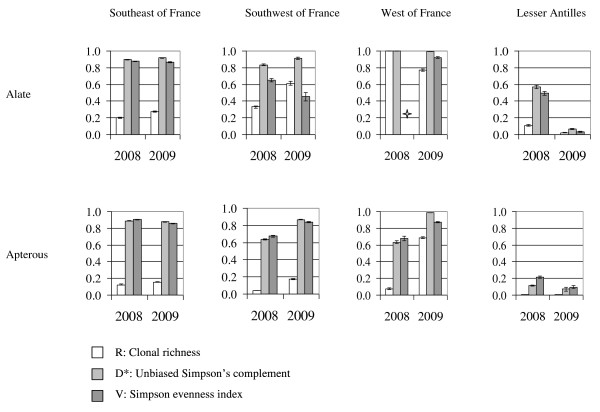
**Clonal diversity of the alate and apterous *A. gossypii *samples collected in 2008 and 2009 in the four melon-growing areas**. R is the index of clonal richness, D* is the unbiased Simpson's complement and is the probability that two individuals chosen at random have different genotypes and can thus be considered as an exact measure of the clonal heterogeneity and V is the Simpson evenness index and is an equitability index that describes the distribution of the components and the relative amount of clones. The confidence intervals were derived from jack-knifing procedures (p = 0.05). ✮ *V *was not estimated because D* was maximal.

The four SE locations had similar values for these indexes for both the alate populations and apterous populations (See additional file 1: figure S1). For example, in the alate populations sampled in 2004, the Simpson's evenness index (*V*) was 0.86 ± 0.05 in Saint-Andiol, 0.83 ± 0.03 in Montfavet and 0.81 ± 0.01 in Eyragues (with a confidence of 95%). No strong differences were observed in these three indexes over the different years in each location. The comparison between the alate and apterous populations showed a significant decrease in the clonal richness of the apterous populations, regardless of the location or the area (See additional file 1: figure S1). We decided to pool the data from the four locations within the SE area for further analyses. The comparison of the four areas (Figure 3) revealed that the lowest diversity of the alate populations was observed within the populations from the LA region and that the highest diversity was observed within the populations from the W region. An intermediate level of diversity was observed in the populations from the SE and SW areas. The clonal richness (*R*) was significantly higher in the SW populations than in the SE populations, while the Simpson's evenness index (*V*) was significantly higher in the populations sampled from the SE area than in the populations sampled from the SW area (no overlapping intervals of confidence at 5%, Figure 3). In all of the cases, a significant decrease in the clonal richness was observed from the alate to the apterous populations (Figure 3).

### Genetic structure of the *A. gossypii *populations

We investigated the aphid genetic structure using AMOVA analyses, considering the effects of the morph (alate *vs*. apterous), the year of sampling, the growing area and the location (Table 2). More than 75% of the variance was distributed within the populations for each of the factors. The only factor that had a significant effect among the groups was the growing-area factor (*P *< 10^-5^), which explained 20% of the variance. The AMOVA analysis confirmed the absence of a significant difference of genetic structure within the populations from the SE region. Therefore, we pooled the samples from all of the SE locations. To search for clustering of the MLGs found in this study, we analysed the MLGs along with the 44 MLGs previously described and assigned to host races (Carletto et al. 2009). Using a Bayesian structuring program, the likeliest numbers of the genetic clusters were K = 2 and K = 4 (See additional file 2: figure S2 for graphics shown in a).

**Table 2:** AMOVA results for the microsatellite data analysis of *A. gossypii *populations

Source of variation		**d.f**.	SS	Variance components	Percentage of variation	*P*
Aphid morph	Among morphs	1	80.58	-0.02	-0.90	0.75
	Among populations	27	3895.73	0.44	18.79	< 10^-5^
	Within populations	9555	18379.12	1.92	82.11	< 10^-5^
Year of sampling	Among years	4	824.57	0.03	1.24	0.32
	Among populations	24	3151.74	0.41	17.29	< 10^-5^
	Within populations	9555	18379.12	1.92	81.47	< 10^-5^
Growing area	Among areas	3	3117.03	0.51	20.05	< 10^-5^
	Among populations	25	859.27	0.10	4.07	< 10^-5^
	Within populations	9555	18379.12	1.92	75.88	< 10^-5^
SE location	Among locations	3	156.11	0.02	0.73	0.14
	Among populations	13	363.75	0.09	4.15	< 10^-5^
	Within populations	5359	10680.51	1.99	95.12	< 10^-5^

*A. gossypii *populations are grouped according to the aphid morph, year of sampling, growing area or SE location.

**Figure 4:**
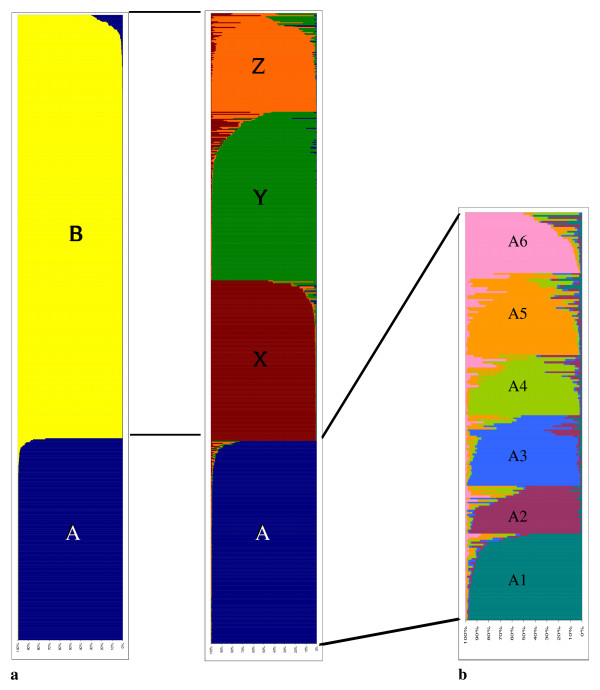
**Results from the Structure program showing the estimated proportion of assignment of the multilocus genotypes**. a. Assignment of the 632 multilocus genotypes to the clusters with K = 2 and K = 4 and b. assignment of the 203 multilocus genotypes of cluster A into 6 sub-clusters.

When K = 2, 203 MLGs of the 596 that were detected in our samples were assigned to cluster A and represented only 5% of the aphids, and 393 MLGs were assigned to cluster B, accounting for 95% of the aphids (Figure 4a). The *F_st _*value between the clusters was 0.254. None of the MLGs that were grouped into cluster A had been described before. When K = 4, cluster A still consisted of the 203 MLGs, but cluster B was divided into three sub-clusters (Figure 4a). Cluster X consisted of 133 MLGs, representing only 4% of the individuals; cluster X also encompassed MLGs that had been previously collected on *Solanum *spp., pepper, cotton, citrus, strawberry and *Hibiscus *spp. plants. Cluster Y contained 163 MLGs, representing 73.5% of the individuals sampled and 11 MLGs that were characteristic of aphids from the *Cucurbitaceae *host race. Therefore, cluster Y was called the cucurbit cluster. Cluster Z, containing 97 MLGs representing 18% of the individuals sampled, contained NM1 and NM1-like MLGs that also characterised aphids from *Cucurbitaceae *hosts along with MLGs that were previously identified on cotton (Ivo) and *Hibiscus *spp. (Hib4) hosts. Therefore, cluster Z was called the NM1 cluster.

We analysed the MLGs that were assigned to cluster B and those that were assigned to cluster A separately using the same parameters of the Structure program. The number of genetic sub-clusters that best fitted the data was K = 3 for cluster B (See additional file 2: figure S2 for graphics shown in b), and the structure was identical to the structure described above for all of the MLGs when K = 4 (Figure 4a). The number of genetic sub-clusters within cluster A was K = 6 (See additional file 2: figure S2 for graphics shown in c), and the 203 MLGs were quite uniformly distributed in the 6 sub-clusters (Figure 4b).

**Figure 5:**
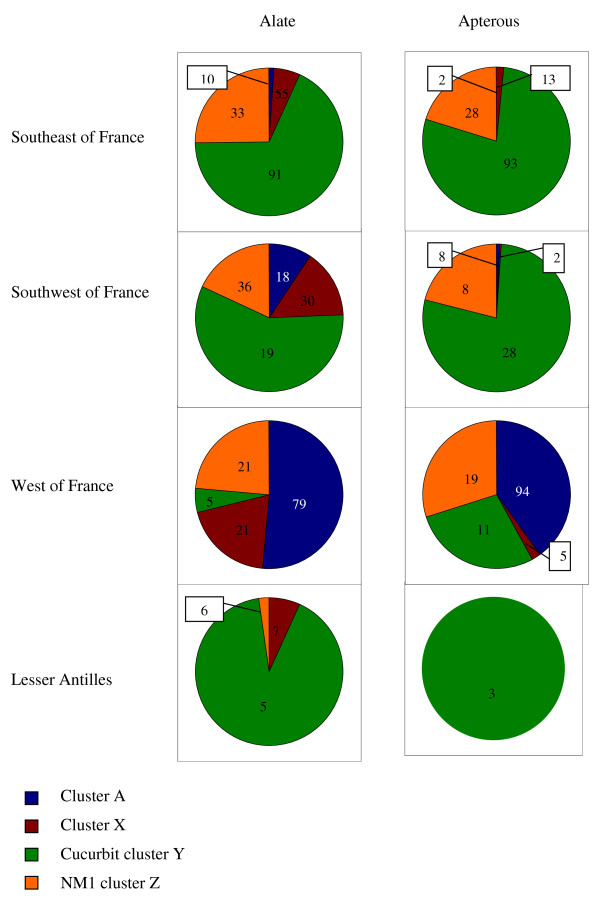
**Distribution of the alate and apterous *A. gossypii *samples according to the genetic clusters**. The proportion of individuals is represented by sectors and the numbers of multilocus genotypes is shown in each sector in the four melon-growing areas according to the division into four genetic clusters.

We considered the distribution of the alate and apterous individuals according to the existence of the four genetic clusters A, X, Y and Z, within each of the four geographic areas (Figure 5). In the SE region, 70% of the alate individuals and 80% of the apterous individuals were assigned to cluster Y (the cucurbit cluster). In the SW region, 60% of the alate individuals and 80% of the apterous individuals were also assigned to the cucurbit cluster. In both the SE and SW areas, 20 to 25% of the individuals, either alate or apterous, belonged to cluster Z (the NM1 cluster). MLGs characteristic of cluster A were present among the alate individuals in the SW region (10%). In the W region, 5% of the alate and 28% of the apterous individuals had an MLG assigned to the cucurbit cluster, and 24 and 30% of the alate and apterous individuals, respectively, had an MLG assigned to the NM1 cluster; approximately 50% of the individuals, regardless of the morph, were assigned to cluster A. In the LA region, most of the alate and apterous individuals had an MLG assigned to the cucurbit cluster (91 to 100%), and these MLGs were not observed in the other growing areas. In all of the areas, 10 to 20% of the alate individuals but almost none of the apterous individuals exhibited an MLG from cluster X (containing MLGs that were characteristics of host races other than *Cucurbitaceae*). Moreover, in all of the areas, the number of individuals with an MLG assigned to the cucurbit cluster or to the NM1 cluster (known for its ability to colonise *Cucurbitaceae*) was significantly higher in the apterous populations than in the alate populations (Chi-square test, *P *< 0.0001). Notably, although the percentage of apterous individuals belonging to the *Cucurbitaceae *host race was equivalent in the SE and SW areas, the diversity among the cucurbit MLGs was considerably higher (Chi-square test, *P *< 0.0001) in the SE region (n = 93) than in the SW region (n = 28) (Figure 5).

### Tracking the genetic fingerprints of sexual reproduction

We examined the genetic variability of the alate populations to track the genetic fingerprints of sexual reproduction. The dataset was completed by the MLGs that were observed within only the apterous samples. We calculated three parameters: the Hardy-Weinberg equilibrium (HWE), the linkage disequilibrium (LD) and the *F_is _*index. To reduce the Wahlund effect, we split the populations a) according to their cluster membership (A, X, Y or Z) and their area (SE, SW, W or LA) (Tables 3) or according to the sub-clusters (A1 to A6) for the individuals assigned to cluster A (Table 4).

**Table 3:** Genetic characteristics of the alate *A. gossypii *populations

Clusters	Areas	N	G	HW	DL	*F_is_*
A	SE	12	10	5/7	13/28	0.610
	
	SW	22	21	3/8	6/28	0.346
	
	W	182	172	0/8	21/28	0.451

X	SE	84	61	1/8	27/28	0.310
	
	SW	32	32	4/8	7/28	0.229
	
	W	37	37	5/8	6/28	0.321
	
	LA	24	7	0/7	20/28	-0.027

Y, the cucurbit cluster	SE	819	130	0/8	27/28	-0.232
	
	SW	135	38	1/8	27/28	-0.180
	
	W	17	13	4/8	18/28	0.079
	
	LA	316	6	0/8	28/28	-0.694

Z, the NM1 cluster	SE	301	40	0/8	28/28	-0.451
	
	SW	43	23	0/8	28/28	0.189
	
	W	55	35	0/8	28/28	0.565
	
	LA	8	6	3/8	25/28	0.051

Number of individuals (N), number of multilocus genotypes (G), number of loci in Hardy-Weinberg equilibrium (HW), number of pairs of loci in linkage disequilibrium (DL), and *F_is _*values across all of the loci in the alate *A. gossypii *populatio*ns *according to the sampling areas in France and four genetic clusters

**Table 4:** Genetic characteristics of the alate *A. gossypii *populations in the sub-clusters

Clusters	N	G	HW	DL	*F_is_*
A1	46	43	2/8	9/28	0.434
A2	24	24	3/6	3/28	0.274
A3	38	35	2/8	12/28	0.335
A4	36	30	5/7	8/28	0.078
A5	41	41	3/7	7/28	0.244
A6	31	30	3/8	5/28	0.259

Number of individuals (N), number of multilocus genotypes (G), number of loci in Hardy-Weinberg equilibrium (HW), number of pairs of loci in linkage disequilibrium (DL), and *F_is _*values across all of the loci in the alate *A. gossypii *populations according to the six genetic sub-clusters within cluster A

The fingerprints of obligate parthenogenesis were departure from the HW equilibrium due to excess heterozygotes, frequent LD and a negative *F_is _*value. These characteristics were observed in the LA region subpopulations in cluster X, in the SE area subpopulation in cluster Z (NM1) and in all of the subpopulations of cluster Y (cucurbit) except for the W subpopulation. The fingerprints of panmictic sexual reproduction were frequent HWE, infrequent LD and a positive *F_is _*value close to 0. The only subpopulation with these characteristics was the W subpopulation in cluster Y. The fingerprints of sexual reproduction by inbreeding were a departure from HWE due to heterozygote deficiency, frequent LD and a positive *F_is _*value close to 1. These fingerprints were observed in the SE area subpopulation in cluster X, the SW and W area subpopulations in cluster Z and the W area subpopulation in cluster A. We observed a fourth situation with a frequent HWE, rare LD and *F_is _*values ranging from 0.23 to 0.61 for the SW and W area subpopulations assigned to cluster X and the SE and SW area subpopulations assigned to cluster A. When cluster A was split into six sub-clusters, HWE was observed for several loci (p > 0.05), and the number of significant LD was low (Table 4). For the other loci, we observed a heterozygote deficiency that was significant at a level of 5% but not of 1%. Moreover, the *F_is _*values across the loci varied from 0.13 to 0.43, suggesting the occurrence of sexual reproduction with a varying level of inbreeding in these subpopulations.

## Discussion

The spring populations of the cotton-melon aphids visiting newly planted melon crops exhibited an unexpectedly high level of genetic diversity that contrasted with the very low diversity characterising the host-specialised populations of this aphid species. The host races identified in *A. gossypii *were dominated by a few asexual clones [22], as a result of host selection, clone competition and pest-management practices [22,34,35]. Because of the use of a novel sampling strategy, the present study illustrated *in natura *host-plant-selection pressure by showing the great differences in genetic diversity between the spring-migrant populations (alate aphids) and the melon-infesting populations (apterous offspring of the alate aphids). Moreover, an analysis of the genetic composition of these alate and apterous populations in four geographic regions suggested differences in their life-history strategies and questioned the common assertion that *A. gossypii *is an anholocyclic species in most of its distribution area, including Europe [36].

### A strikingly high genetic variability

Using the same set of microsatellite markers, previous genetic analyses of more than 3600 *A. gossypii *individuals discriminated only 44 MLGs. Each of these individuals corresponded to an apterous aphid taken from a different colony on vegetable crops of the *Cucurbitaceae, Solanaceae, Malvaceae, Rutaceae *and *Rosaceae *families in different countries [25,26]. Almost every MLG was present in multiple copies, and 70% of the aphids were distributed in only 4 MLGs that were each associated with a particular crop. The level of genetic diversity detected in the present study was more than ten times higher: 596 MLGs were identified from approximately 4800 aphids sampled exclusively on melon crops in France and the Lesser Antilles (Figure 1). This difference may be explained by the time of sampling. In the present study, we collected the aphids as soon as they migrated into the melon parcels at the very beginning of the crop season, while in the previous studies, apterous aphids were collected in infested crops from colonies that had already undergone the selective filter of plant choice and acceptance. The majority of the 596 MLGs (70%) identified in the present study were present as single copies, and only 4 of the MLGs had a frequency greater than 5% (Table 1). This distribution was roughly the same in the two studied morphotypes, alate and apterous. Nevertheless, the percentage of individuals that exhibited unique or rare MLGs was only 22% among the alate aphids and 9% among the apterous aphids. Therefore, although a high genetic variability was found, this study showed that the number of clones that recurred over time and space were dominated by only a dozen MLGs (Figure 2).

The analysis of the 596 MLGs revealed four genetic clusters (Figure 4). Three of the clusters had already been identified in previous studies, although the genetic diversity observed within each of them was much higher in the present study. These three clusters contained the MLGs that characterised the aphid clones that were specialised on cultivated plants. Cluster Y contained the MLGs that defined the Cucurbitaceae host race (as defined by [22]); these MLGs were found in more than 70% of the whole dataset. Cluster Z contained some MLGs that specialised on *Cucurbitaceae *(NM1) and some that specialised on *Malvaceae*; these MLGs were found in 18% of all of the individuals. Cluster X contained MLGs from the *Solanaceae *and cotton host races; these MLGs were found in less than 5% of the aphids. In contrast, cluster A contained 203 MLGs that had never been observed in previous studies. However, these new MLGs represented less than 5% of the sampled aphids. The hypothesis that the aphids bearing these MLGs may have originated from a broad array of wild host plants is supported by the huge host-plant spectrum of *A. gossypii*, which consists of more than 900 plant species over 119 botanical families [28].

### Host-plant selection

Previous studies demonstrated the existence of host races in the species *A. gossypii*. These hyper-specialised clones are found all over the world on the same crops. Because these aphids specialise on annual crops, they are submitted to strong demographic bottlenecks, and they have to produce alate morphs at least twice during the annual cycle, first at the end of the crop season to disperse on refuge plants and again at the beginning of the next season to migrate back to the host crops. Therefore, the settlement of a new colony challenges alate aphids because they may have to make long flights to find a suitable host plant [37]. Alate aphids have been shown to use visual and olfactory cues to alight [38-40]. Only a few studies have been conducted on winged-aphid olfactory responses to plant volatiles [41]. Studies of flight behaviour suggested that landing frequency increased in traps that released host-plant volatiles compared to empty control traps. However, it was not demonstrated that winged aphids landed significantly more frequently on their specific host plants. Moreover, the ability to recognise a suitable host plant from a distance, which should be selectively advantageous for a clone, has never been shown *in natura*. In the present study, we observed alate individuals that were obviously not specialised on *Cucurbitaceae *on melon crops, *i.e*., individuals belonging to the clusters A and X. This finding suggests that olfactory cues are unlikely to be decisive *in natura *for host recognition, which probably occurs only after landing and plant probing. To test this hypothesis, we compared the diversity and the genetic structure of alate and apterous populations on melon crops at the very beginning of the crop season. As expected, the clonal richness was significantly lower in the apterous samples than in the alate samples in all of the studied fields (Figure 3 and See additional file 1: figure S1). Moreover, this decrease in diversity was mainly due to a loss of the MLGs belonging to the clusters X and A. In the south of France (the SE and SW regions) and in the Lesser Antilles (LA), these MLGs, which represented up to a quarter of the alate populations, almost completely disappeared in the apterous populations (Figure 5). This tendency was confirmed in the west of France, where the proportion of the MLGs of the clusters A and X decreased from approximately three quarters in the alate population to one third in the apterous population. This finding argues in favour of host-plant recognition by alate aphids after probing. The clones that were not adapted to melons might lay a few larvae before taking off again. Indeed, in *A. fabae*, studies investigating the relationships between stylet penetration, *i.e*., probing, and parturition showed that parturition is stimulated by chemical cues that are detected before contact with the phloem [37]. However, these few nymphs were not adapted to feed and therefore to reproduce on melon, as exemplified by the fact that none of them developed colonies. All of the colonies that were observed corresponded to *Cucurbitaceae*-specialised MLGs from the clusters Y or Z. Our results clearly demonstrate that the melon plant acts as a selective filter against the reproduction of non-specialised individuals.

### Geographical structure

The genetic diversity of the populations on the melon crops was geographically structured, as revealed by the AMOVA (Table 2, among groups *P *< 10^-5 ^when groups are growing areas) and pairwise *F_st _*analysis (and See additional file 3: table S3). This structure could be related to the characteristics of the four agrosystems under study. *Cucurbitaceae *are one of the main vegetable crops cultivated in France based on the surface area used for production, more than 20,000 ha in mainland France, equally distributed among three regions (southeast, southwest and west), and 4,000 ha in the Lesser Antilles (Guadeloupe and Martinique).

The differentiation was especially strong between the aphid populations from the Lesser Antilles and those from France. The genetic diversity in the Lesser Antilles appeared to be unique and very low; the majority of the individuals exhibited the same MLG belonging to the cucurbit cluster (Table 1, Figures 3, 5). Obviously, these particular MLGs do not result from a recent introduction of aphids through the high level of commercial exchanges between France and the French West Indies. The introduction might be related to the triangular trade between Europe, Africa and America in the 17^th ^century, or even earlier, to the colonisation by native Americans who travelled from the region of Venezuela through the whole chain of the West Indies approximately 4,000 years ago up to the arrival of Europeans.

As mentioned by Zepeda-Paulo et al. (2010) [42], the evolutionary processes associated with aphid introductions into new geographical areas have not been extensively studied, but as suggested by Normark (2003) [43], a temperate aphid lineage transported to the tropics would likely switch to obligate parthenogenesis. Therefore, the very low genetic diversity observed in the melon aphid populations in the Lesser Antilles could result from the introduction of a small number of clones, of which only those able to adapt to the selective pressures of the new environmental conditions could rapidly multiply and disperse.

When considering the populations from France, a geographical structure of the genetic diversity was detected (*F_st _*analysis, See additional file 3: table S3). In the SE region, we observed that 70% of the individuals were assigned to the cucurbit cluster (Figure 5). These individuals exhibited 91 out of the 163 MLGs identified within this cluster, which was the highest clonal richness that we observed within the cucurbit cluster. In the SW region, 60% of the individuals fell within the cucurbit cluster, and those individuals displayed only 19 of the MLGs in that cluster. In the W region, only 5% of the individuals were assigned to the cucurbit cluster and shared 5 MLGs. These genetic differences could be related to differences in the importance of *Cucurbitaceae *crops, both across seasons and years in these three production areas. From an historical point of view, the melon crop has been of significance for many decades in the SE and SW regions, while in the W region, melons have been grown on a large scale for only ten years. Therefore, cucurbit-adapted MLGs have been favoured and selected for longer in the south than in the western region. In addition, in the SE region, *Cucurbitaceae *crops are grown from January to the end of the fall, either under cover or in open fields, while in the SW region, the plants are restricted to open fields in the spring and summer. The availability of the *Cucurbitaceae *resource throughout the aphid annual cycle varies considerably among regions, and that variation could explain the differences in the level of genetic diversity of the melon-adapted populations. In the SE area, the time window after which the *Cucurbitaceae *resource is no longer available is very small. Therefore, the *Cucurbitaceae*-specialised populations do not experience a harsh bottleneck during the winter, allowing for the maintenance of slightly divergent MLGs produced by mutation. The high genetic diversity observed in the *Cucurbitaceae*-specialised populations in the SE region probably results from both long-term selection and weak demographic bottlenecks during the winters. Another significant difference lies in the proportion of the alate aphids belonging to cluster A, which was ten times higher in the samples from the SW region (10%) than in the SE region (1%). This difference might be due to a difference in the uniformity of the agricultural environment between the two regions, resulting in a lower abundance of the overwintering colonies of specialised *Cucurbitaceae *clones in the SW region, and therefore, reducing the alate spring population size.

### Reproductive mode

Parthenogenesis is much more common among insect pests than related non-pest insect species, most likely because agricultural environments are stable with an abundance of resources [44]. The same genotypes may be continuously favoured by selection, leading to a selective advantage of some parthenogenetic lineages over a sexual population. Almost 600 MLGs were detected in this study, of which 28% were observed more than once over the entire sample of approximately 4800 aphids. These repeated MLGs characterised 90% of the individuals, most of which belonged to the cucurbit cluster. The values of the genetic indices shown in Tables 3 and 4 (HW, DL and *F_is_*) strongly suggest that these individuals correspond to obligate parthenogenetic lineages, in accordance with previous genetic studies [22,25,26,45]. Sexual-reproduction fingerprints were, however, detected in the *Cucurbitaceae*-specialised population collected in the W region (Table 3). Although this result needs to be confirmed in a larger sample, it is supported by biological studies that revealed that a few clones sampled from *Cucurbitaceae *hosts were able to induce oviparous female and/or male morphs during conditions of shortened daylight periods [45,46], Carletto (unpublished data). Moreover, as mentioned earlier, the relative heterogeneity of the *Cucurbitaceae *agrosystem in the W region could explain the maintenance of sexual reproduction in cucurbit-specialised populations.

Another *Cucurbitaceae*-specialised MLG, NM1, was found in 76% of the individuals from cluster Z. This MLG also corresponded to a parthenogenetic lineage. To date, this MLG has been reported only in aphid samples from *Cucurbitaceae *crops in France [22,47]. The NM1 MLG was largely predominant in the SE population where it accounted for the negative value of the *F_is _*index (Table 3). The frequency of this MLG in the SW and W regions was less than 50%. In addition to NM1, cluster Z contained 59 unique MLGs and 37 MLGs present more than once (See additional file 4: table S4). A genetic analysis comparing the populations from the SW and W regions suggested that sexual reproduction occurred in these populations (Table 3). The positive values of *F_is _*argued in favour a high level of inbreeding but they could also be artificially inflated by pooling different reproductive units (Wahlund effect) or because of a mix of asexual and sexual lineages.

Cluster X was composed of 133 MLGs, of which 115 were unique, and the 18 MLGs left existed in two or three copies (See additional file 4: table S4). As shown in Figure 5, there was no redundancy in the MLGs among the different areas, and the sum of the MLGs found in every region was equal to 133. The genetic indices suggested the occurrence of sexual reproduction with inbreeding in populations from the SE, SW and W regions. The 133 MLGs belonging to cluster X were grouped with MLGs detected over several years in France and collected from cultivated plant hosts, such as *Solanum *spp., pepper, citrus, strawberry and *Hibiscus *spp. (Carletto, 2009). The origin of the new MLGs remains unknown.

Cluster A was composed of 203 MLGs from 229 individuals, indicating that almost every aphid had a different MLG (See additional file 4: table S4). Most of these genotypes (85%) were detected in populations from the W region (Figure 5) and they were quite uniformly distributed in the six sub-clusters of the A cluster (Figure 4b). The genotypes detected in populations from SE and SW regions were also distributed in the six sub-clusters. The genetic analyses within the populations belonging to the sub-clusters A suggested that sexual reproduction occurred with a high level of inbreeding in these populations (Table 4). As mentioned earlier, these aphids are assumed to colonise wild plant species. The uncultivated plant compartment could be considered as a heterogeneous and disturbed environment, in which the maintenance of sexuality confers advantages, while crop plants correspond to simplified and uniform environments that favoured a few host-adapted asexual lineages [48,49].

## Conclusions

In conclusion, the genetic survey of the alate aphids revealed that sexual reproduction occurs in French populations of *A. gossypii*, challenging the commonly shared view that the melon aphid reproduces exclusively by obligate parthenogenesis even in temperate regions [36]. Approximately 75% of the alate aphids collected in France were characterised by a dozen multi-copy MLGs and corresponded to parthenogenetic lineages that are highly specialised on *Cucurbitaceae*. The availability and abundance of the host-plant resource likely selected for the parthenogenetic reproductive mode of plant-adapted genotypes [44]. Population genetic structure analyses conducted on two pests of cereal crops, *Sitobion avenae *[50] and *Rhopalosiphum padi *[51], revealed that the populations were distributed in two clusters according to their reproductive mode (*i.e*., sexual *vs*. asexual lineages). Populations of *R. padi *were not genetically differentiated according to host-plant origin [52], whereas in *S. avanae*, a much higher proportion of sexual lineages were found on uncultivated host plants [16]. Distinct clusters corresponding to populations with a different reproductive mode were also evidenced within the host race *nicotianae *of *M. persicae *[24]. Our study demonstrated that the differentiation of the *A. gossypii *populations depended on both the reproductive strategy and host plant. The Bayesian clustering analysis was used to first separate the populations into two groups. One group consisted of cluster A, with individuals assumed to reproduce sexually and to develop on wild plants, and the other group consisted of cluster B, in which asexual lineages co-existed with a few sexual lineages. The sub-clustering of B led to the identification of the cluster Y, containing mainly parthenogenetic lineages specialised on *Cucurbitaceae*, and the clusters X and Z, which likely displayed a mixed reproductive strategy. The high level of diversity detected in the spring alate aphids was generated by the reproduction of the sexual lineages. However, this huge diversity was counter-selected by the melon plants, as evidenced by the much lower diversity found in the apterous offspring. Moreover, none of the non-specialised offspring was able to develop a colony. We cannot exclude that some of the parthenogenetic lineages from the *Cucurbitaceae *host race are androcyclic, producing males and parthenogenetic females or are intermediate, investing into both sexual and parthenogenetic reproduction. Their sexual morphs could migrate to the primary host to mate either with an unrelated MLG or with a cucurbit MLG (assortative mating). Indeed, the cucurbit-specialised clone C9, which was found in high frequency in *Cucurbitaceae *crops all over the world [22], could be intermediate as it was shown to develop sexual morphs in the lab when submitted to induction conditions [45]. New asexual lineages could result from the occasional mating between females from sexual lineages and males produced by asexual lineages that could transmit asexuality genes [53]. Therefore, the evolution of *A. gossypii *as a pest of melon crops would depend on the balance between the gain of genetic diversity and the loss of host-adapted gene combinations resulting from sexual reproduction. The evolution of parthenogenetic lineages in the *Cucurbitaceae *host race also relies on the selection of new mutants exhibiting a higher fitness in response to pest-management practices and to the clonal amplification of these new variants [35].

## Methods

### Melon-growing area descriptions and sampling locations

Four melon-growing areas were considered in this study - in the southeast, southwest, and west of France and in Guadeloupe in the Lesser Antilles (Figure 1). In the southeastern region (SE), the aphids were sampled at Eyragues (43°49'50'' N, 4°49'50'' E), Montfavet (43°56' 44" N, 4°51' 52"E), Saint-Andiol (43°50'07'' N, 4°56'40'' E) and Aramon (43°54'59'' N, 4°43'08'' E). This area is characterised by a Mediterranean climate, with a wet winter/dry summer seasonality of precipitation. The southwestern (SW) and western (W) regions of France have a more temperate climate, under the influence of the Gulf Stream. The aphids were sampled at Moissac (44°07'13.9''N, 01°03'17.5''E) (SW), which has early springs, long warm summers and mild winters, and in Angers (47°28'38.7''N, 0°36'49.2''E) (W), which is located in the northern part of the melon-growing area, with mild and humid weather throughout the year. In the Lesser Antilles (LA), where sugarcane and banana are the major crops, the aphids were sampled in the melon-crop area at Petit-Canal (16°24'04'' N, 61°29'0.9''O) on Grande-Terre Island (Guadeloupe). This limestone plateau regularly experiences severe droughts, with almost no temperature variation throughout the year.

### Aphid sampling and DNA analysis

The aphids were sampled over five years (2004, 2006, 2007, 2008 and 2009) in seven melon fields (no insecticide was applied excepted in Petit-Canal where Basudine^® ^was applied once a few days before transplanting). The fields contained plants without genetic resistance to aphids. One population was defined as a sample collected in a field a given year and with a given morph, alate or apterous. Melons were transplanted in fields at the end of March in the LA and in May in the SE, SW and W regions of France. We collected 15 to 228 alate morphs per field during the first three weeks after planting; the aphids were collected from the melon plants and constituted a sample of the spring migrants that alighted on the melon crops. Sampling of apterous morphs, which consisted of the spring migrants offspring, started three weeks after planting and was conducted over four to five weeks. We collected 80 to 491 apterous morphs per field. Each apterous aphid was collected either as an isolated individual or as one individual representing the colony that was produced by a single asexual founding mother. Each colony that was sampled was tagged to avoid any re-sampling.

The DNA of 4,792 individual aphids was extracted using a 5% (w/v) Chelex resin solution, as described by Fuller *et al. *(1999). The DNA amplifications at eight microsatellite loci specific to the *A. gossypii *genome [54] were performed in two PCR reactions, as described by [22]. The allele size at each of the loci was identified by comparison with a molecular size standard using the software GeneMapper v3.7 (Applied Biosystems, Foster City, CA); a multilocus genotype (MLG) was subsequently assigned to each aphid.

### Data analyses

#### Genetic diversity

Three indices were computed for each population as proposed by [55], considering the alate and apterous samples separately, to estimate the genetic diversity and the level of aggregation of identical genotypes. The index of clonal richness (*R*) was computed as $R=\frac{G-1}{N-1}$, where *G *is the number of distinct genotypes in the population and N is the total number of sampled individuals from the population. The unbiased Simpson's complement (D*) was calculated as $D^{*}=1-\overset{G}{\underset{i}{\sum }}\frac{n_{i}\left(n_{i}-1\right)}{N\left(N-1\right)}$, where *n_i _*is the number of occurrences of genotype *i *and *N *is the total number of sampled individuals from the population. This index can be interpreted as the probability that two individuals chosen at random have different genotypes and can thus be considered as an exact measure of the clonal heterogeneity. The Simpson evenness index (*V*) was computed as $V=\frac{\left(D^{*}-D_{\min}\right)}{\left(D_{\max}-D_{\min}\right)}$, where $D_{\min}=\left[\frac{\left(2N-G\right)\times \left(G-1\right)}{N^{2}}\right]\times \frac{N}{\left(N-1\right)}$ and $D_{\max}=\frac{\left(G-1\right)}{G}\times \frac{N}{\left(N-1\right)}$. *V *is an equitability index that describes the distribution of the components and the relative amount of clones. *R *and D* range from 0, if all of the individuals in the population are clones (*G *= 1), to 1, when each sampled individual in the population bears a distinct genotype (*G *= *N*). The Simpson evenness index (*V*) varies between 0 and 1, which represent extreme skew and evenness, respectively. We used a jack-knifing procedure to estimate the standard error of R, D* and *V: *for each sample set, we recomputed the 3 indices, leaving out one observation at a time from the sample set. From this new set of observations, an estimate for the variance of the indices was calculated, and thereafter, their confidence interval at 5% was calculated [56].

#### Genetic structure

We used hierarchical analyses of molecular variance, AMOVA, and pairwise *F_st _*tests [57] in the Arlequin version 3.1 software [58] to test for genetic structure variation related to the aphid morph, growing area, sampling year or location. The significance was tested using 100,000 permutations.

#### Assignment to host races

In our assignment procedure, we will refer to the four host races that have been unambiguously identified (*Cucurbitaceae*, cotton, *Solanum *and pepper) from numerous samples of *A. gossypii *collected over several years on a large geographical scale from annual crops of different plant families [22]. We assigned each MLG observed in our samples to the appropriate cluster as follows. The MLGs identified in the different populations were analysed together with the 44 MLGs previously assigned by Carletto et al. (2009) [22] using the Bayesian program Structure [59]. We used the admixture model with a burn-in of 500,000 and a subsequent Markov Chain of 250,000 iterations. Ten replicate runs for each value of a putative number of clusters *K *(varying from 1 to 30) were compared to check the consistency of the estimates and to determine the likeliest number of genetic clusters. We paid particular attention to those MLGs that clustered with the 12 cucurbit reference MLGs that were defined by Carletto et al. (2009) [22].

#### Tracking the genetic fingerprints of sexual reproduction

The genetic fingerprints of sexual reproduction were studied in the spring-migrant populations, *i.e*., the alate samples, collected in the early season in the melon fields. We computed three parameters: departures from the Hardy-Weinberg equilibrium (HWE) for each locus, the linkage disequilibrium (LD) for each pair of loci and the *F_is _*value using the exact tests (p = 0.05) available in the Arlequin version 3.1 program, considering repeated genotypes [60]. The MLGs that were only observed in apterous populations were added because these MLGs were necessarily present in the alate mother population. The *F_is _*index can be thought of as a measure of the identity of alleles within individuals relative to the identity of alleles randomly drawn from two different individuals issued from the same subpopulation. The *F_is _*value is interpreted in terms of the deviation from random mating caused by the breeding system of the organism under study: for a strictly clonal population, *F_is _*= -1; for a strictly sexual population, *F_is _*= 0; and for a strictly inbreeding population, *F_is _*= +1. To reduce the Wahlund effect, the samples collected in each of the growing areas were divided according to the genetic clusters evidenced using the Structure program. The calculations of the HWE, LD and *F_is _*were conducted on these sub-samples.

## Competing Interests

The authors declare that they have no competing interests.

## Authors' contributions

ST carried out the acquisition, analysis and interpretation of the data and drafted the manuscript. NB conceived and designed the study and was involved in the acquisition and interpretation of the data and in drafting the manuscript. FV was involved in the acquisition and interpretation of the data and in revising the manuscript. All author read and approved the final manuscript.

## Supplementary Material

Additional file 1 **Figure S1**. Clonal diversity of the alate and apterous *A. gossypii *samples collected from 2004 to 2009 in the four locations in southeastern France. R is the index of clonal richness, D* is the unbiased Simpson's complement and is the probability that two individuals chosen at random have different genotypes and can thus be considered as an exact measure of the clonal heterogeneity and V is the Simpson evenness index and is an equitability index that describes the distribution of the components and the relative amount of clones. The confidence intervals derive from jack-knifing procedures (p = 0.05). Click here for file

Additional file 2 **Figure S2**. Mean values of the ln likelihood and delta K obtained for ten simulations of each K after Structure analysis completed with a. all of the MLGs, b. the MLGs assigned to cluster B and c. the MLGs assigned to cluster A. Click here for file

Additional file 3 **Table S1**. Genetic differentiation between the *A. gossypii *populations sampled in three areas of France and in the Lesser Antilles according to the pairwise *F_st_*. Click here for file

Additional file 4 **Table S2**. Number of individuals (N), number of multilocus genotypes (G), and the number of unique MLGs (g) in the alate (Al) and apterous (Ap) *A. gossypii *populations sampled in France and the Lesser Antilles, grouped according to the clusters. Click here for file
