# Supplementary material for: What do spring migrants reveal about sex and host selection in the melon aphid?
Source: BMC Evol Biol. 2012 Apr 3;12:47. doi: 10.1186/1471-2148-12-47 (PMC3368726; doi:10.1186/1471-2148-12-47)
Supplement: Additional file 1 — Figure S1. Clonal diversity of the alate and apterous A. gossypii samples collected from 2004 to 2009 in the four locations in southeastern France. R is the index of clonal richness, D* is the unbiased Simpson's complement and is the probability that two individuals chosen at random have different genotypes and can thus be considered as an exact measure of the clonal heterogeneity and V is the Simpson evenness index and is an equitability index that describes the distribution of the components and the relative amount of clones. The confidence intervals derive from jack-knifing procedures (p = 0.05). [file 1471-2148-12-47-S1.DOC]

Aramon St-Andiol Montfavet Eyragues

Alate

Apterous

**Figure A:** Clonal diversity of the alate and apterous *A. gossypii* samples collected from 2004 to 2009 in the four locations in southeastern France. R is the index of clonal richness, D* is the unbiased Simpson’s complement and is the probability that two individuals chosen at random have different genotypes and can thus be considered as an exact measure of the clonal heterogeneity and V is the Simpson evenness index and is an equitability index that describes the distribution of the components and the relative amount of clones. The confidence intervals derive from jack-knifing procedures (p=0.05).
