# Supplementary material for: What do spring migrants reveal about sex and host selection in the melon aphid?
Source: BMC Evol Biol. 2012 Apr 3;12:47. doi: 10.1186/1471-2148-12-47 (PMC3368726; doi:10.1186/1471-2148-12-47)
Supplement: Additional file 2 — Figure S2. Mean values of the ln likelihood and delta K obtained for ten simulations of each K after Structure analysis completed with a. all of the MLGs, b. the MLGs assigned to cluster B and c. the MLGs assigned to cluster A. [file 1471-2148-12-47-S2.DOC]

**a**

**b**

**c**

**Figure B:** Mean values of the ln likelihood and delta K obtained for ten simulations of each K after Structure analysis completed with a. all of the MLGs, b. the MLGs assigned to cluster B and c. the MLGs assigned to cluster A.
