# Supplementary material for: What do spring migrants reveal about sex and host selection in the melon aphid?
Source: BMC Evol Biol. 2012 Apr 3;12:47. doi: 10.1186/1471-2148-12-47 (PMC3368726; doi:10.1186/1471-2148-12-47)
Supplement: Additional file 3 — Table S1. Genetic differentiation between the A. gossypii populations sampled in three areas of France and in the Lesser Antilles according to the pairwise Fst. [file 1471-2148-12-47-S3.DOC]

**Table A**: Genetic differentiation between the *A. gossypii* populations sampled in three areas of France and in the Lesser Antilles according to the pairwise *F_st_*.

|  | Southeast of France | Southwest of France | West of France |
| --- | --- | --- | --- |
| Southwest of France | 0.07963*** |  |  |
| West of France | 0.18077*** | 0.13795*** |  |
| Lesser Antilles | 0.25632*** | 0.30611*** | 0.36417*** |

*** P value < 0.0001
