# Supplementary material for: What do spring migrants reveal about sex and host selection in the melon aphid?
Source: BMC Evol Biol. 2012 Apr 3;12:47. doi: 10.1186/1471-2148-12-47 (PMC3368726; doi:10.1186/1471-2148-12-47)
Supplement: Additional file 4 — Table S2. Number of individuals (N), number of multilocus genotypes (G), and the number of unique MLGs (g) in the alate (Al) and apterous (Ap) A. gossypii populations sampled in France and the Lesser Antilles, grouped according to the clusters. [file 1471-2148-12-47-S4.DOC]

**Table B**: Number of individuals (N), number of multilocus genotypes (G), and the number of single MLGs (g) in the alate (Al) and apterous (Ap) *A. gossypii* populations sampled in France and the Lesser Antilles, grouped according to the clusters.

|  | Cluster A | | | Cluster X | | | Cluster Y | | | Cluster Z | | |
| --- | --- | --- | --- | --- | --- | --- | --- | --- | --- | --- | --- | --- |
|  | Al | Ap | T | Al | Ap | T | Al | Ap | T | Al | Ap | T |
| N | 120 | 109 | 229 | 157 | 31 | 188 | 1215 | 2308 | 3523 | 376 | 476 | 852 |
| G | 107 | 99 | 203 | 123 | 19 | 133 | 111 | 112 | 163 | 72 | 50 | 97 |
| g | 95 | 91 | 181 | 112 | 29 | 115 | 56 | 49 | 75 | 52 | 33 | 59 |
